# Improving patient recruitment to randomised trials can be cost-effective: A case-study of dexamethasone from the RECOVERY trial

**DOI:** 10.1371/journal.pone.0314593

**Published:** 2025-04-01

**Authors:** Athanasios Gkekas, Sarah J. Ronaldson, Adwoa Parker, David J. Torgerson

**Affiliations:** 1 York Trials Unit, Department of Health Sciences, University of York, York, United Kingdom; 2 York Health Economics Consortium, York, United Kingdom; Stanford University School of Medicine, UNITED STATES OF AMERICA

## Abstract

**Background:**

The RECOVERY trial assessed the effectiveness of treatments on preventing severe outcomes from COVID-19 disease in hospitalised patients from 176 NHS hospitals. Clinical benefits of Dexamethasone were observed for hospitalised COVID-19 patients. About 15% of all eligible patients were recruited into the trial. Had patient recruitment been higher the study would have been completed more rapidly.

**Aim:**

To estimate the cost-effectiveness of improving recruitment to the RECOVERY trial from 15% to 50%, by employing or redeploying two research nurses to each hospital participating in the RECOVERY trial. The analysis is restricted to the evaluation of Dexamethasone versus No Dexamethasone.

**Methods:**

A decision tree model was developed to estimate the cost-effectiveness of Dexamethasone, against No Dexamethasone. Probability, utility, and cost inputs were used for each pathway and treatment. Then, a cost-utility analysis of clinical practice post-RECOVERY trial (83% Dexamethasone, 17% No Dexamethasone) versus previous clinical practice (100% No Dexamethasone) was undertaken; this analysis was aggregated at the population level and the cost of employing or redeploying two research nurses at each hospital was added, to estimate the cost-effectiveness of faster recruitment to the RECOVERY trial.

**Results:**

Faster recruitment to the RECOVERY trial could have generated an incremental net benefit of £13,955,476 related to the evaluation of Dexamethasone against No Dexamethasone, thus highlighting the magnitude of the foregone incremental net benefit due to not adopting a more cost-effective clinical practice (83% Dexamethasone, 17% No Dexamethasone) earlier. The findings remain robust following variations in the model’s parameters, with a 85% and 94% probability of faster recruitment being cost-effective given a cost-effectiveness threshold of £20,000 and £30,000 per Quality Adjusted Life Year respectively.

**Conclusion:**

Slow recruitment to randomised trials can have huge implications for healthcare systems as a result of not introducing a more cost-effective treatment earlier through faster patient recruitment.

## Introduction

### Background

When the COVID-19 pandemic struck the United Kingdom one of the initial responses was to rapidly implement the RECOVERY trial to evaluate the effectiveness of potential treatments on preventing severe outcomes from COVID-19 disease in hospitalised patients [1]. Patients admitted to one of the 176 participating hospitals with suspected or PCR-test confirmed SARS-Cov-2 infection received one of the five treatments: dexamethasone, hydroxychloroquine, lopinavir–ritonavir, azithromycin, or usual care alone. RECOVERY was designed as a randomised controlled, open-label, adaptive, multi-armed platform trial. The recruitment began on March 19th, 2020, and by June 8th, 2020, 11,303 COVID-19 patients consented and underwent randomisation, of whom 9,355 (83%) could receive dexamethasone. Of the 9,355 eligible COVID-19 patients, 6,425 participated in the comparison of dexamethasone plus usual care vs usual care alone for assessing their effectiveness in reducing 28-day mortality [1]. The results of the dexamethasone arm from the RECOVERY trial were announced on June 16th, 2020 [2].

Most benefits of dexamethasone were observed for COVID-19 patients who received invasive mechanical ventilation or/and non-invasive ventilation (oxygen only) [1]. The incidence of COVID-19-related death in the dexamethasone plus usual care group was significantly lower compared to the usual care group (no dexamethasone), with the rate ratio being 0.64 (95% Confidence Interval (CI): 0.51,0.81), for patients who received invasive ventilation, and 0.82 (95% CI: 0.72,0.94), for patients who received non-invasive ventilation as their most intensive treatment for COVID-19 [1].

The dexamethasone results were produced rapidly despite the average recruitment rate across the participating hospitals being 15%, with recruitment ranging from 3% to 80% per hospital [2]. Slow recruitment can lead to underpowered studies, delays in the conduct and dissemination of research, ethical issues, and costly extensions of randomised trials [3]. A review of 388 trials funded by the National Institute for Health Research (NIHR) from 1997 to 2020 found 33% of trials required an extension, and 20% revised their recruitment target downward, demonstrating that slow recruitment is a frequent challenge in the conduct of randomised trials [4].

A study estimated the potential clinical benefits of faster recruitment to the RECOVERY trial in reducing COVID-19 mortality in the UK [5]. Originally, with the 15% recruitment rate, patient recruitment ended on June 8^th^, 2020. Instead, with a 50% recruitment rate, the recruitment target could have been reached by April 1^st^, 2020 [5]. Thus, the trial’s results could have been disseminated on April 9^th^, instead of June 16^th^, allowing an earlier update to clinical practice. This could have potentially saved at least 2,620 additional lives [5].

#### Aims and hypothesis

Our study aims to estimate the cost-effectiveness of faster recruitment to the RECOVERY trial, focusing on the use of dexamethasone as a treatment for hospitalised COVID-19 patients. By considering the RECOVERY Collaborative Group’s findings [1] and the estimates on the effectiveness of faster recruitment to the RECOVERY trial related to the evaluation of dexamethasone [5], we explore the value of increasing the recruitment rate from 15% to 50% through a potentially effective recruitment strategy, from the economic perspective of a national healthcare system.

Such a strategy could be the employment or redeployment of two Band 5 research nurses to each National Health Service (NHS) hospital affiliated with the study. Assuming it takes an hour on average to speak to each patient about the study, each nurse could recruit up to six patients daily. Given a 17% exclusion rate in the study [1] and an assumed refusal rate of 20%, this figure would drop to 3.78 patients a day. With 352 research nurses, up to 1,331 patients could be recruited daily, thus reaching the recruitment target of 11,303 patients in 8.5 working days.

## Methods

### Cost-effectiveness of faster recruitment to the RECOVERY trial

We develop a decision tree model and follow a multi-step approach to estimate the cost-effectiveness of faster to the RECOVERY trial. This analysis is also stratified by age groups (45–64, 65–74, and 75+).

Step 1: For each treatment arm, each pathway’s QALYs and costs are multiplied by the corresponding probabilities to calculate the expected QALYs and costs.
Step 2: We calculate the incremental QALYs and costs of Dexamethasone and No Dexamethasone to estimate the cost-effectiveness of Dexamethasone. Results are presented in terms of the incremental cost-effectiveness ratio (ICER), i.e. the incremental cost per QALY gained, and the incremental net benefit (INB) using a £20,000 cost-effectiveness threshold.
Step 3: If dexamethasone was (cost-) effective for reducing the incidence of COVID-related mortality, the updated clinical practice would encourage the 10-day provision of dexamethasone to hospitalised COVID patients. However, only 83% of hospitalised COVID patients can receive Dexamethasone as 17% of them are expected to have contraindications to dexamethasone [1], hence the remaining (17%) patients receive usual care only (No Dexamethasone). Therefore, we estimate the cost-effectiveness of updated practice (83% Dexamethasone, 17% No Dexamethasone) versus previous clinical practice (100% No Dexamethasone), by weighting appropriately the expected QALYs and costs from Step 1, for different age groups and the population overall, with the findings presented as ICERs and INBs.
Step 4: With a 15% recruitment rate, 6,980 hospitalised patients are estimated to have originally benefited from the updated practice up to mid-July 2020 (15% recruitment rate) [5]. With a 50% recruitment rate instead, 77,310 hospitalised patients are estimated to have benefited from the updated practice up to mid-July 2020 [5]. The incremental cost of faster recruitment is the salary costs of 352 research nurses (i.e. two nurses per site). To break down the number of benefited patients and the costs of nurses into age groups, we use estimates for the proportion of COVID-19 inpatients at NHS hospitals using daily averages of the age distribution from 12/10/2020 to 04/01/2022 [6].
Step 5: The incremental QALYs and costs of improving recruitment from 15% to 50%, by recruiting two full-time nurses to each hospital, are calculated by multiplying the expected QALYs and costs of updated practice (Step 3), with the number of patients that would have benefited from it with a 50% recruitment rate or the number of patients that benefited from it with a 15% recruitment rate (Step 4). The INB of faster recruitment to the RECOVERY trial and the ICER are the study’s outcomes.

The study follows the reporting standards outlined from the Consolidated Health Economic Evaluation Reporting Standards (CHEERS) statement. The CHEERS checklist for the study can be found in S1 Table. Our study uses secondary and already published data, thus there was no requirement for ethical approval.

### Decision problem

**Fig 1:**
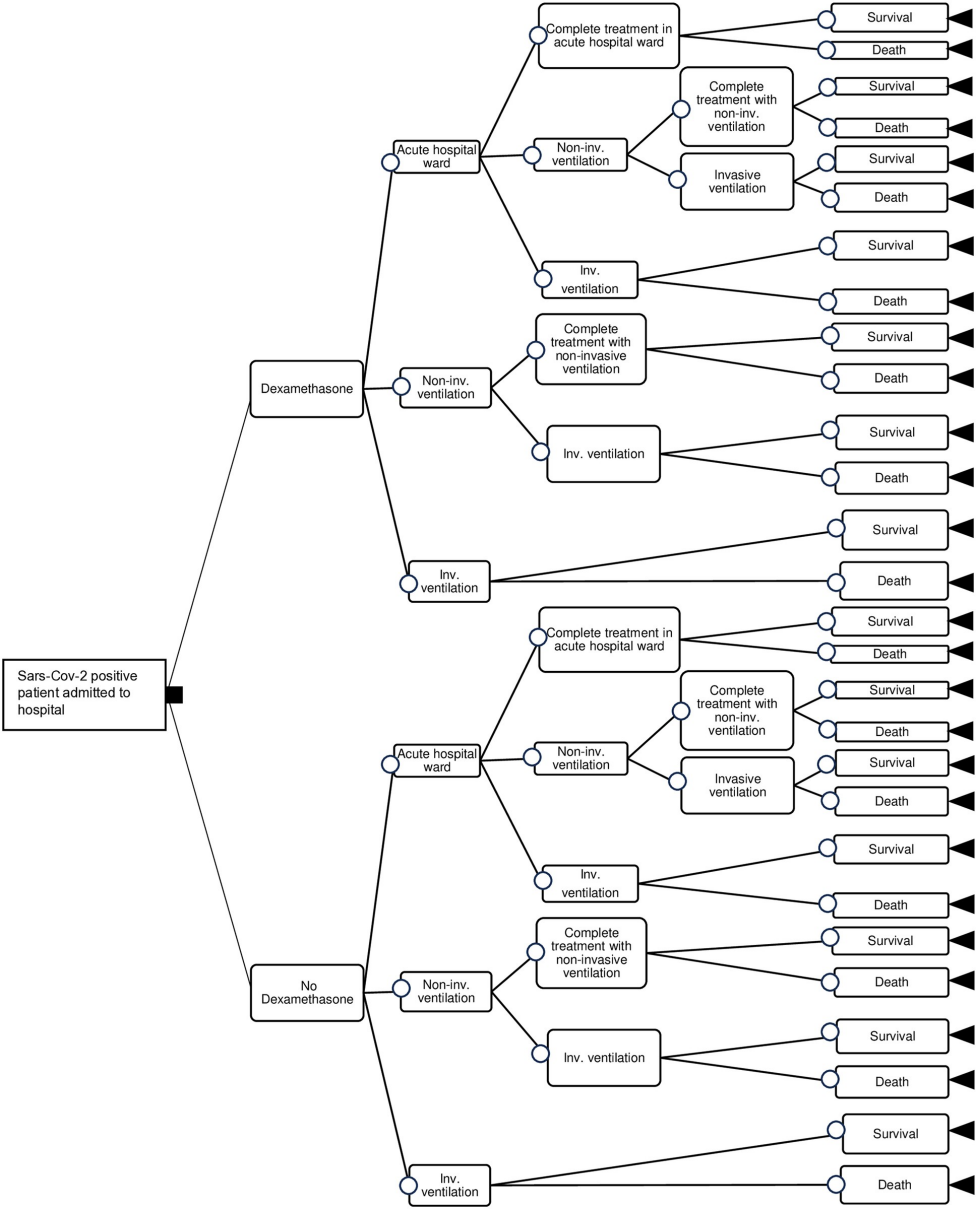
Decision tree.

A decision tree model is set up in Microsoft Excel (Fig 1) to evaluate the cost-effectiveness of faster recruitment to the RECOVERY trial, through a prior cost-utility analysis of Dexamethasone versus No Dexamethasone for the clinical management of hospitalised COVID-19 patients. Published studies that estimated the cost-effectiveness of dexamethasone and other treatments evaluated from the RECOVERY trial, in relation to COVID-19 disease, have also applied decision tree methods [7, 8].

A patient, irrespective of their age, enters the model with SARS-Cov-2 infection and is admitted to an NHS hospital. This patient receives treatment either in an acute hospital ward with no ventilation support, or in an Intensive Care Unit (ICU) with non-invasive (oxygen only) or invasive (mechanical) ventilation. Such set of treatments is referred to as *usual care*. Since the trial does not distinguish between patients who received oxygen only with or without non-invasive ventilation [1], we assume that patients requiring oxygen only, but not invasive mechanical ventilation, are admitted to the ICU and receive non-invasive ventilation. The patient may also receive 6mg of dexamethasone per day for up to 10 days following hospital admission, i.e., in the dexamethasone plus usual care group (Dexamethasone). This setting aligns with the RECOVERY trial’s design [1]. Therefore, the patient may enter one of the following:

- Dexamethasone plus usual care (defined as *Dexamethasone)*: 6mg of dexamethasone per day plus usual care for up to 10 days, followed by usual care alone if hospitalised for longer, or;
- Usual care *(defined as No Dexamethasone)*: Usual care only for as long as needed.

### Clinical pathways and decision tree

The decision tree’s 28 pathways (14 related to Dexamethasone and 14 related to No Dexamethasone), presented in Table 1, are developed according to the clinical outcomes of the RECOVERY trial [1]. The pathways associated with survival from COVID-19 disease also capture the possibility of a patient experiencing long COVID for up to six months after SARS-Cov-2 infection. The probability inputs for long COVID are differentiated for patients who receive invasive ventilation to those who do not [9]. Long COVID is incorporated in the model and defined as persistent fatigue, with severity similar to myalgic encephalomyelitis/chronic fatigue syndrome (ME/CFS). The probability inputs are presented in Table 2. S1 File presents in detail the estimation of the probability inputs related to the RECOVERY trial’s findings [1].

**Table 1:** Clinical pathways.

Pathway (Dexamethasone arm)	Pathway (No Dexamethasone arm)	Initial admission	Course of treatment	Outcome
P1	P15	Acute hospital ward	No further admission to ICU.	Survival
P2	P16	Acute hospital ward	No further admission to ICU.	Death
P3	P17	Acute hospital ward	Further admission to ICU with non-invasive ventilation (i.e. oxygen treatment). No receipt of invasive mechanical ventilation.	Survival
P4	P18	Acute hospital ward	Further admission to ICU with non-invasive ventilation (i.e. oxygen treatment). No receipt of invasive mechanical ventilation.	Death
P5	P19	Acute hospital ward	Further admission to ICU with non-invasive ventilation (i.e. oxygen treatment), then to ICU with invasive mechanical ventilation.	Survival
P6	P20	Acute hospital ward	Further admission to ICU with non-invasive ventilation (i.e. oxygen treatment), then to ICU with invasive mechanical ventilation.	Death
P7	P21	Acute hospital ward	Further admission to ICU with direct provision of invasive mechanical ventilation.	Survival
P8	P22	Acute hospital ward	Further admission to ICU with direct provision of invasive mechanical ventilation.	Death
P9	P23	ICU with non-invasive ventilation (i.e. oxygen treatment)	No receipt of invasive mechanical ventilation at ICU.	Survival
P10	P24	ICU with non-invasive ventilation (i.e. oxygen treatment)	No receipt of invasive mechanical ventilation at ICU.	Death
P11	P25	ICU with non-invasive ventilation (i.e. oxygen treatment)	Further receipt of invasive mechanical ventilation at ICU.	Survival
P12	P26	ICU with non-invasive ventilation (i.e. oxygen treatment)	Further receipt of invasive mechanical ventilation at ICU.	Death
P13	P27	ICU with invasive mechanical ventilation.	No further upgrade.	Survival
P14	P28	ICU with invasive mechanical ventilation.	No further upgrade.	Death

**Table 2:** Probability inputs.

**Probabilities related to types of usual care available at admission**	**Source**		**All groups**
P(acute hospital ward at admission)	[1]		0.239
P(non-invasive ventilation received at admission)	[1]		0.604
P(invasive ventilation received at admission)	[1]		0.157
**Probabilities of clinical outcomes and admissions to more intensive types of usual care during hospitalisation with COVID-19**	**Source**	**Dexamethasone**	**No Dexamethasone**
P(death|acute hospital ward)	[1]	0.166	0.132
P(non-invasive ventilation|acute hospital ward)	[1]	0.032	0.044
P(invasive ventilation|acute hospital ward)	[1]	0.010	0.029
P(survival|acute hospital ward)	[1]	0.784	0.794
P(death| non-invasive ventilation)	[1]	0.206	0.217
P(invasive ventilation|non-invasive ventilation)	[1]	0.079	0.107
P(survival|non-invasive ventilation)	[1]	0.715	0.676
P(death| invasive ventilation)	[1]	0.293	0.414
P(survival| invasive ventilation)	[1]	0.707	0.586
**Probabilities related to long COVID**	**Source**		**All groups**
P(long COVID; acute hospital ward, non-invasive ventilation)	[9]		0.128
P(long COVID, invasive ventilation)	[9]		0.262

### Perspective

The study adopts the perspective of the English National Health Service (NHS) which considers the resource use of primary and secondary healthcare service, as well as prescription costs.

### Time horizon

A time horizon of one year is adopted. We recognise that, due to the recent emergence of the COVID-19 pandemic, there is uncertainty with respect to several long-term outcomes; hence outcomes such as reinfection with Sars-Cov-2, rehospitalisation with COVID-19, and mortality following rehospitalisation with COVID-19, are not incorporated. The nature of this uncertainty arises from the potential emergence of immunity-resistant variants of SARS-Cov-2 and the future public health response to the trajectory of the COVID-19 pandemic. At the time of the RECOVERY trial, when the original strain of SARS-Cov-2 was transmitted across the UK, there were no COVID-19 vaccines available for use. However, the study still considers longer-term outcomes such as recovery from COVID-19 disease for a patient having received invasive mechanical ventilation versus a patient not having received invasive ventilation, and the risk of long COVID for up to six months following infection with SARS- Cov-2.

### Health outcomes

To estimate health outcomes for each pathway, data on the utility weights of their relevant health states are collected. Each pathway’s relevant utility weights are then multiplied by the corresponding day weights (relative to a year of 365.25 days) to estimate their yearly Quality-Adjusted Life Years (QALYs).

In terms of estimating the utilities of health states, we collect population-wide and age-specific utility weights related to the average health state of the population residing in England [10]. Moreover, we consider the average health states of the 45–64, 65–74 and 75+ age groups, as they have the highest risk of hospital admission along with/due to SARS-Cov-2 infection [11]. Then, we obtain age-independent disutility weights to estimate the utilities of additional health states, including SARS-Cov-2 infection, hospitalisation in an acute ward, and hospitalisation along with non-invasive and invasive ventilation support [12]. In addition, an age-independent disutility weight for long COVID, identical to that of ME/CFS [13] is incorporated. For each pathway, we estimate population-wide and age-specific utility weights by subtracting the corresponding disutility weights from the baseline utility weights. The corresponding inputs are shown in Table 3.

**Table 3:** Utility and length of stay (LoS) inputs.

**Input**	**Source**	**Utility weight (45 to 64 age group)**	**Utility weight (65 to 74 age group)**	**Utility weight (75+ age group)**	**Utility weight (Population total)**	**Disutility weight (all age groups)**
Average healthy state	[10]	0.849	0.785	0.734	0.856	N/A
COVID-19 Infection	[10, 12]	0.579	0.515	0.464	0.586	0.27
Hospitalisation (Acute ward, no ventilation received)	[10, 12]	0.469	0.405	0.354	0.476	0.11 (plus SARS-Cov-2 infection)
Hospitalisation (Non-invasive ventilation received)	[10, 12]	0.219	0.155	0.104	0.226	0.36(plus SARS-Cov-2 infection)
Hospitalisation (Invasive ventilation received)	[10, 12]	0.019	0	0	0.026	0.56 (plus SARS-Cov-2 infection)
Death	N/A	0	0	0	0	N/A
Long COVID (post-COVID syndrome)	[10, 13]	0.559	0.495	0.444	0.566	0.29
**Input**	**Source**			**Figure**		
Days of COVID-19 infection	[14]			6.7		
Days of long COVID (from the beginning of infection with Sars-Cov-2)	[9]			182.63		
Maximum hospitalisation days (acute hospital ward, survival)	[15]			9.4		
Maximum hospitalisation days (from acute hospital ward to ICU)	[15]			2.0		
Maximum hospitalisation days (acute hospital ward, death)	[15]			8.3		
Maximum hospitalisation days (non-invasive ventilation, survival)	[12]			12.58		
Maximum hospitalisation days (non-invasive ventilation, death)	[12]			9.41		
Maximum hospitalisation days (invasive ventilation, survival)	[15]			24.5		
Maximum hospitalisation days (invasive ventilation, death)	[15]			15.8		

In terms of obtaining the appropriate day weights, estimates of the duration of Sars-Cov-2 infection before admission to hospital [14], hospitalisation days associated with each pathway [12, 15], and the duration of long COVID following survival [9] are collected, all of which are divided by 365.25 days to obtain the corresponding weights. The remaining days each year are treated as COVID-free days, and the corresponding day weights are attached and multiplied by the appropriate utility weight of the average health state. Long COVID is incorporated into the estimation of QALYs by multiplying its likelihood of (not) occurring (see Table 2), with the corresponding disutility weight.

We estimate hospitalisation days for each pathway by the best study available to date regarding the length of stay (LoS) in NHS hospitals due to COVID-19 disease [15]. However, since there is no distinction between non-invasive ventilation and invasive ventilation in the study’s estimates, we also consider a US study to differentiate the LoS between non-invasive ventilation and invasive ventilation [12]. For clinical pathways, where a clinical course involves a less invasive and a more invasive treatment, we assume that the maximum expected number of days with the less invasive treatment are needed before a patient is transferred to a more invasive treatment. It was possible to derive differentiated hospitalisation days for those who survived and those who died, with those dying from COVID-19 staying in hospital for fewer days [15].

### Costs

Unit daily costs are included for: non-ICU treatment (code: XC07Z) [16]; ICU treatment (codes: XC06Z, DZ37A) [16]; and 6mg of dexamethasone [17]. Costs are reported in 2020 price levels.

As the NHS Reference costs do not distinguish between non-invasive and invasive ventilation [16], the cost of invasive ventilation is calculated via a multiplication of the cost of non-invasive ventilation reported from the NHS Reference Costs by a multiplier, i.e. 1.258, from a review that has estimated the impact of mechanical ventilation on the daily costs of ICU care [18]. Where costs are available in different price levels, a UK GDP deflator is used to adjust the figures to 2020 levels [19]. Daily costs related to hospitalisation are multiplied by the expected number of hospitalisation days corresponding to each pathway [12, 15].

If a patient survives from COVID-19 disease, they have a chance of getting long COVID. In this case, we assume they may need up to two GP consultations in a six-month period for screening and treatment purposes. Therefore, we also include the unit costs of two GP appointments [20].

The Dexamethasone arm faces the same costs as the No Dexamethasone arm, but with the addition of the daily cost of receiving 6mg of dexamethasone for up to 10 days. 28 Dexamethasone 2mg tablets have an NHS indicative price of £2.10, 50 tablets have an NHS indicative price of £3.75, and 100 tablets have an indicative price of £8.93 [17], implying that three tablets of 2mg (leading to the daily intake of 6mg of dexamethasone) cost £0.23, £0.13, and £0.27 correspondingly. The median value of £0.23 is considered for the baseline cost-utility analysis, whereas the other two prices are considered for sensitivity analysis.

The corresponding cost inputs are reported in Pound Sterling (GBP) and presented in Table 4.

**Table 4:** Cost inputs.

Input	Source	Cost (£)
Unit cost of GP appointment (long COVID patients)	[20]	£39.23
Daily cost of staying in acute hospital ward	[16, 19]	£748.41
Daily cost of non-invasive ventilation (oxygen)	[16, 19]	£1,394.23
Daily cost of invasive ventilation	[16, 19]	£1,753.94
Daily cost of providing 6mg of dexamethasone	[17]	£0.23
Salary cost of Research Nurse (Band 5 maximum point)	[20, 21]	£39,840.77

The annual salary cost of a Band 5 research nurse is also reported, although it will not be considered as a cost input for the cost-utility analysis of dexamethasone; instead, it will be considered for the subsequent cost-utility analysis of faster recruitment to the RECOVERY trial as it is related to the recruitment strategy. The salary cost (i.e. £39,841) reflects the annual gross wage of a Band 5 research nurse (i.e. £30,615 [21]) plus the salary oncosts (i.e. employer’s national insurance contributions plus 20.68% of gross salary for employer’s contribution to superannuation [20]).

### Discounting of health effects and costs

As all relevant costs and health effects are expected to occur within the first year of a patient being hospitalised with/due to Sars-Cov-2 infection, there is no discounting of future health effects and costs.

### Probabilistic sensitivity analysis (PSA)

We apply a random sampling approach of 10,000 iterations of input parameter values across all the generated distributions to produce distributions of incremental QALYs and incremental costs, and therefore a distribution of the INB of faster recruitment to the RECOVERY trial, via Excel VBA. Under this distribution, we also estimate the 95% confidence interval of the INB of faster recruitment to the RECOVERY trial. The probability, utility and cost inputs used for PSA, together with their distributions, means and standard errors, are presented in S2 Table. The distributions of the incremental QALYs and the incremental costs are graphically presented on a cost-effectiveness plane, whereas a cost-effectiveness acceptability curve (CEAC) is also available.

## Results

### Cost-effectiveness of Dexamethasone as a treatment for COVID-19 hospitalised patients

Dexamethasone is cost-effective relative to No Dexamethasone, with the ICER being £1,236, which is significantly lower than the £20,000 cost-effectiveness threshold. Using the same threshold, the INB of Dexamethasone, against No Dexamethasone, is £481. Dexamethasone remains cost-effective across age groups. The summary of the cost-utility analysis of Dexamethasone against No Dexamethasone, for the population and age groups, is shown in Table 5.

**Table 5:** Cost-utility analysis of Dexamethasone against No Dexamethasone.

**Treatment Group**	**Expected QALYs**	**Expected costs**	**Incremental QALYs**	**Incremental costs**	**Incremental net benefit (£)** *	**ICER** *
Dexamethasone	0.621	£18,757.84	0.025	£31.66	£476.02	£1,247.27
No Dexamethasone	0.596	£18,726.18				
Patients aged 45–64 years old						
**Treatment Group**	**Expected QALYs**	**Expected costs**	**Incremental QALYs**	**Incremental costs**	**Incremental net benefit (£)**	**ICER**
Dexamethasone	0.568	£18,757.84	0.023	£31.66	£434.44	£1,358.53
No Dexamethasone	0.545	£18,726.18				
Patients aged 65–74 years old						
**Treatment Group**	**Expected QALYs**	**Expected costs**	**Incremental QALYs**	**Incremental costs**	**Incremental net benefit (£)**	**ICER**
Dexamethasone	0.529	£18,757.84	0.022	£31.66	£403.12	£1,456.39
No Dexamethasone	0.507	£18,726.18				
Patients aged 75+ years old						
**Treatment Group**	**Expected QALYs**	**Expected costs**	**Incremental QALYs**	**Incremental costs**	**Incremental net benefit (£)**	**ICER**
Dexamethasone	0.627	£18,757.84	0.026	£31.66	£480.67	£1,235.94
No Dexamethasone	0.601	£18,726.18				
Population (total)						

*All Incremental net benefit and ICER calculations used a cost-effectiveness threshold of £20,000.

### Cost-effectiveness of updated clinical practice

The updated clinical practice (i.e., where 83% of patients treated with Dexamethasone and 17% of patients treated with No Dexamethasone) is cost-effective compared with previous clinical practice (i.e., 100% of patients treated with No Dexamethasone), with the ICER being £1,236, which is significantly lower than the £20,000 cost-effectiveness threshold. Using the same threshold, the INB of updated clinical practice is £398. The updated clinical practice remains cost-effective across all age groups. The summary of the cost-utility analysis of updated clinical practice against previous clinical practice, for the population and age groups, is shown in Table 6.

**Table 6:** Cost-utility analysis of updated clinical practice against previous clinical practice.

**Clinical practice**	**Expected QALYs**	**Expected costs**	**Incremental QALYs**	**Incremental costs**	**Incremental net benefit (£)** *	**ICER** *
Updated clinical practice	0.617	£18,752.38	0.021	£26.20	£393.98	£1,247.27
Previous clinical practice	0.596	£18,726.18				
Patients aged 45–64 years old						
**Clinical practice**	**Expected QALYs**	**Expected costs**	**Incremental QALYs**	**Incremental costs**	**Incremental net benefit (£)**	**ICER**
Updated clinical practice	0.564	£18,752.38	0.019	£26.20	£359.57	£1,358.53
Previous clinical practice	0.545	£18,726.18				
Patients aged 65–74 years old						
**Clinical practice**	**Expected QALYs**	**Expected costs**	**Incremental QALYs**	**Incremental costs**	**Incremental net benefit (£)**	**ICER**
Updated clinical practice	0.525	£18,752.38	0.018	£26.20	£333.65	£1,456.39
Previous clinical practice	0.507	£18,726.18				
Patients aged 75+ years old						
**Clinical practice**	**Expected QALYs**	**Expected costs**	**Incremental QALYs**	**Incremental costs**	**Incremental net benefit (£)**	**ICER**
Updated clinical practice	0.623	£18,752.38	0.021	£26.20	£397.83	£1,235.94
Previous clinical practice	0.601	£18,726.18				
Population (total)						

*All Incremental net benefit and ICER calculations used a cost-effectiveness threshold of £20,000.

Updated clinical practice: 83% receive Dexamethasone and 17% receive No Dexamethasone; previous clinical practice: 100% receive usual care.

### Cost-effectiveness of faster recruitment to the RECOVERY trial

Achieving faster recruitment, by employing or redeploying two research nurses at each NHS hospital, would be cost-effective, with the ICER being £10,641. Under the £20,000 cost-effectiveness threshold, the population-level INB of faster recruitment to the RECOVERY trial is £13,955,476. Achieving faster recruitment remains cost-effective across all age groups. The summary of the cost-utility analysis of 50% recruitment rate against 15% recruitment rate, for the population as a whole and by age group, is shown in Table 7.

**Table 7:** Cost-utility analysis of faster recruitment to the RECOVERY trial.

**Recruitment Status**	**Incremental QALYs of updated practice**	**Incremental costs of updated clinical practice**	**Incremental QALYs of faster recruitment**	**Incremental costs of improved recruitment**	**Incremental net benefit of improved recruitment (£)** *	**ICER** *
Faster Recruitment	410.364	£4,055,041.46	373.314	£4,008,829.90	£3,457,453.67	£10,738.49
Actual Recruitment	37.050	£46,211.56				
Patients aged 45–64 years old						
**Recruitment Status**	**Expected QALYs**	**Expected costs**	**Incremental QALYs**	**Incremental costs**	**Incremental net benefit (£)**	**ICER**
Faster Recruitment	232.007	£2,497,095.64	211.060	£2,468,638.55	£1,752,561.72	£11,696.38
Actual Recruitment	20.947	£28,457.09				
Patients aged 65–74 years old						
**Recruitment Status**	**Expected QALYs**	**Expected costs**	**Incremental QALYs**	**Incremental costs**	**Incremental net benefit (£)**	**ICER**
Faster Recruitment	456.333	£5,265,326.73	415.1329	£5,205,322.66	£3,097,335.57	£12,538.93
Actual Recruitment	41.200	£60,004.07				
Patients aged 75+ years old						
**Recruitment Status**	**Expected QALYs**	**Expected costs**	**Incremental QALYs**	**Incremental costs**	**Incremental net benefit (£)**	**ICER**
Faster Recruitment	1639.106	£16,049,788.75	1491.118	£15,866,884.13	£13,955,476.42	£10,640.93
Actual Recruitment	147.988	£182,904.62				
Population (total)						

*All Incremental net benefit and ICER calculations used a cost-effectiveness threshold of £20,000.

### Sensitivity analysis

**Fig 2:**
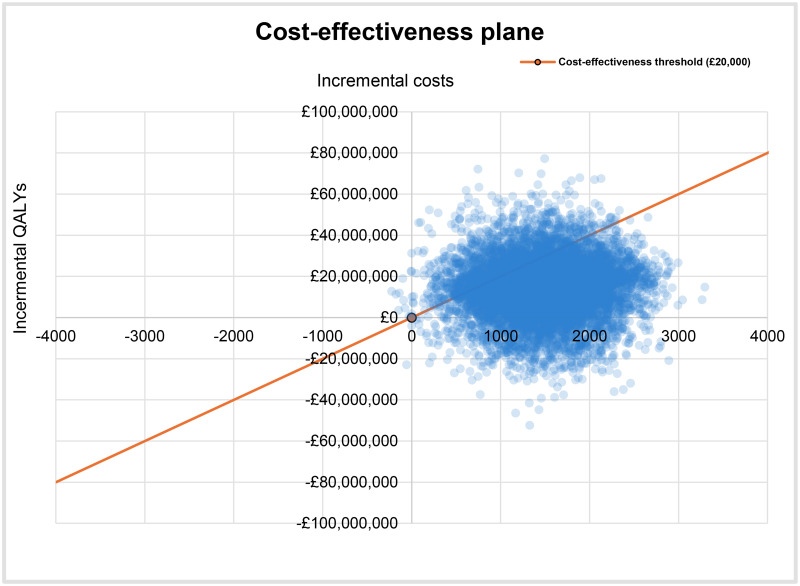
Cost-effectiveness plane.

As the cost-effectiveness plane *in* Fig 2 demonstrates, most random combinations of incremental QALYs and incremental costs lie on the right-hand side of the threshold gradient on the top right and bottom left quadrants, with combinations also observed on the bottom right quadrant. Therefore, by considering the underlying uncertainty, it is highly likely that faster recruitment to the RECOVERY trial is a cost-effective strategy, compared to actual recruitment.

**Fig 3:**
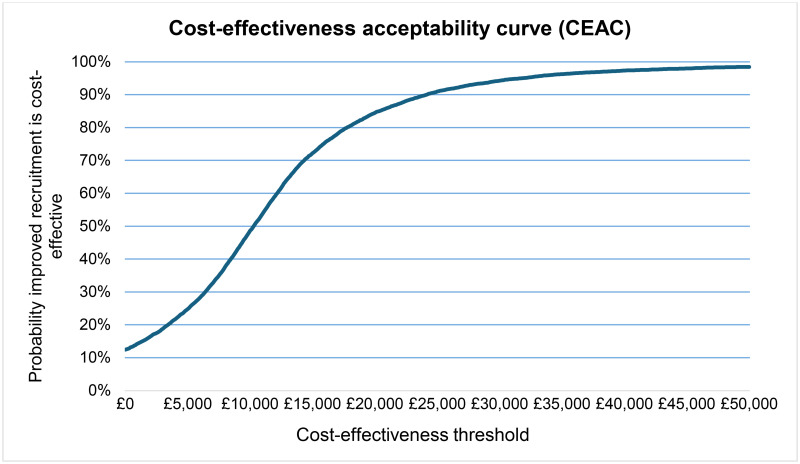
Cost-effectiveness acceptability curve (CEAC).

The probabilistic INB of faster recruitment to the RECOVERY trial is £15,306,802 (95% CI: £14,972,787, £15,640,816). At the £20,000 threshold, the lower limit NICE recommends for the evaluation of health technologies [22], the probability of faster recruitment being cost-effective is 85%. At the £30,000 cost-effectiveness threshold, the upper limit NICE recommends [22], this probability increases to 94%. The cost-effectiveness acceptability curve (CEAC) is shown in Fig 3.

## Discussion

### Summary of findings

Our findings highlight the importance of improving recruitment of patients to RCTs, from the economic perspective of a healthcare system, by considering a noticeable case study related to the COVID-19 pandemic. Improving the recruitment rate of the RECOVERY trial could have generated an incremental net benefit of £13,955,476, thus highlighting the magnitude of the foregone incremental net benefits due to slow recruitment. By employing or redeploying two research nurses to each involved hospital to accelerate recruitment, £10,641 would need to be invested to generate an incremental QALY for COVID-19 hospitalised patients, a figure significantly below the recommended cost-effectiveness threshold of £20,000 per QALY gained [22]. As the NHS is a fixed budget national healthcare system, it is essential for policy makers to prioritise research and resources into improving patient recruitment to randomised trials.

Our sensitivity analysis confirms the robustness of the findings, with the probability of faster recruitment being a cost-effective strategy at 85% and 94% under the £20,000 and £30,000 cost-effectiveness thresholds, respectively.

### Strengths and limitations of the study

This is the first study that highlights how crucial patient recruitment is for improving health outcomes and making clinical practice cost-effective. Whereas the impact of poor recruitment is usually discussed for statistical reasons such as reduced power, this study also demonstrates that the adoption of appropriate recruitment strategies could also be a cost-effective approach in the case of dexamethasone as a treatment for COVID-19 hospitalised patients. In fact, it is highly likely that our INB estimate is underestimated since it is primarily based upon the UK hospitalisation and death data. As the COVID-19 pandemic was affecting the operations of multiple healthcare systems worldwide, a lot more than 2,620 lives would have been saved had the results of the RECOVERY trial been disseminated in April rather than mid-June 2020 and had all countries updated their clinical practices accordingly.

Improving recruitment to randomised trials could be cost-effective in three distinct scenarios. Firstly, since the use of dexamethasone as a treatment for COVID-19 patients increases QALYs at a relatively low incremental cost, more QALYs could have been gained had its implementation been faster through faster recruitment to the RECOVERY trial. Alternatively, if a treatment under evaluation was deemed ineffective, there would be an indirect QALY gain from the earlier identification of an alternative, efficacious treatment. This is facilitated via faster recruitment to trials assessing the (cost-) effectiveness of the former treatment, leading to its premature cessation. Finally, if a treatment under evaluation was found to be harmful, QALYs would fall. In this case, there could be a potential cost gain, as well as a direct QALY gain from the avoidance of a prolonged use of such a treatment. For instance, the RECOVERY trial observed an increase in mortality (albeit not statistically significant) among patients randomised to the hydroxychloroquine arm, which could have led to a reduction in the worldwide prescription of this treatment for COVID-19 [23]. The current analysis did not take these additional benefits into account. Thus, accelerating knowledge about which treatments are ineffective is also important as many of these are costly and have no clinical value.

One of the key limitations of our study lies in the model’s structural assumptions. For instance, we assumed that survived patients would fully recover outside from hospital; whereas this is true for some patients, many of them would remain in acute hospital beds for a smooth recovery. However, the trial’s findings did not provide any information on the likelihood and the length of stay (LoS) of survived and ventilated COVID-19 patients on acute hospital beds [1]. In addition, we assumed that the expected costs of dexamethasone, usual care, updated clinical practice and previous clinical practice are uniform across all age groups due to a lack of age-stratified LoS data applicable to our decision model. Nevertheless, the impact of age on LoS variance is moderate (less than 10%) [15], suggesting that actual cost differences across age groups may be small.

Despite the significance of our findings, employing additional research nurses is not an evidence-based recruitment strategy [3]. Theoretically, however, research nurses could actively support hospitalised COVID-19 patients during their participation into the RECOVERY trial, and effectively communicate the benefits and risks of participating in the trial. Such a support would be valuable given the RECOVERY chief investigator’s remarks, which related slow recruitment to the poor willingness of some patients to enter a trial, and the lack of promotion of the RECOVERY trial to some patients [2]. Furthermore, several NHS hospitals had already achieved recruitment rates of above 50% (i.e. as high as 80%) [2]; thus, employing or redeploying research nurses could have improved the recruitment performance of the most affected hospitals. We also anticipated it could take only 8.5 working days to reach the desirable number of patients recruited to the trial, by adopting this strategy. However, it might also be the case that, at the onset of a global pandemic, employing or redeploying 352 nurses across the UK may not have been feasible. However, it is also possible that, with many elective surgeries and primary care appointments cancelled, such a recruitment strategy would be feasible.

### Direction for future research

Our study is based on an ex-post cost-utility analysis, where the dissemination of a cost-effective treatment was delayed due to slow recruitment. Alternatively, if a treatment was not cost-effective, improving recruitment could still be cost-effective but would have to be modelled differently. As discussed, this study does not explore these alternative situations, and we therefore encourage further research in this area.

With respect to improving patient recruitment to randomised trials, the conduct of Studies Within A Trial (SWATs) is highly suggested, in order for effective patient recruitment strategies to be identified. A SWAT is a “self-contained research study that has been embedded within a host trial with the aim of evaluating or exploring alternative ways of delivering or organising a particular trial process” [24]. Currently, there is limited evidence as to which recruitment strategies could be effective with a high degree of certainty, with open trials, compared to placebo trials, and telephone reminders, compared to no reminders, being promising strategies [3]. Therefore, it is crucial that SWATs of new and existing recruitment strategies be undertaken to achieve more efficient patient recruitment to randomised trials.

## Conclusion

This study highlights the importance of improving patient recruitment to randomised trials from the economic perspective of a national healthcare system. Slow recruitment to the RECOVERY trial led to foregone incremental net benefits, as clinical practice for COVID-19 inpatients could not be updated earlier with dexamethasone, a proven cost-effective treatment. Therefore, it is crucial to identify and adopt effective recruitment strategies that would allow national healthcare systems to benefit from faster patient recruitment to randomised trials.

## Supporting information

S1 Table CHEERS checklist. (DOCX)

S2 Table Inputs for probabilistic sensitivity analysis (PSA). (DOCX)

S1 File Estimation of the input probabilities using the results of the RECOVERY trial. (DOCX)
