# Supplementary material for: Improving patient recruitment to randomised trials can be cost-effective: A case-study of dexamethasone from the RECOVERY trial
Source: PLoS One. 2025 Apr 1;20(4):e0314593. doi: 10.1371/journal.pone.0314593 (PMC11961003; doi:10.1371/journal.pone.0314593)
Supplement: S1 Table — (DOCX) [file pone.0314593.s001.docx]

**S1 Supporting Information. CHEERS Checklist**

**S1 Table. CHEERS Checklist**

| **Topic** | **No.** | **Item** | **Location where item is reported** |
| --- | --- | --- | --- |
| **Title** |  |  |  |
|  | 1 | Identify the study as an economic evaluation and specify the interventions being compared. | Section: “Title” |
| **Abstract** |  |  |  |
|  | 2 | Provide a structured summary that highlights context, key methods, results, and alternative analyses. | Section: “Abstract” |
| **Introduction** |  |  |  |
| **Background and objectives** | 3 | Give the context for the study, the study question, and its practical relevance for decision making in policy or practice. | Sub-Sections: “Background”, “Aims and Hypothesis” |
| **Methods** |  |  |  |
| **Health economic analysis plan** | 4 | Indicate whether a health economic analysis plan was developed and where available. | Not reported; not applicable |
| **Study population** | 5 | Describe characteristics of the study population (such as age range, demographics, socioeconomic, or clinical characteristics). | Section: “Methods”; Sub-Section: “Decision Problem” |
| **Setting and location** | 6 | Provide relevant contextual information that may influence findings. | Section: “Methods”; Sub-Sections: “Decision Problem “and ““Clinical pathways and decision tree” |
| **Comparators** | 7 | Describe the interventions or strategies being compared and why chosen. | Section: “Methods”; Sub-Section:  ” Cost-effectiveness of faster recruitment to the RECOVERY Trial” |
| **Perspective** | 8 | State the perspective(s) adopted by the study and why chosen. | Section: “Methods”; Sub-Section: “Perspective” |
| **Time horizon** | 9 | State the time horizon for the study and why appropriate. | Section: “Methods”; Sub-Section: “Time horizon” |
| **Discount rate** | 10 | Report the discount rate(s) and reason chosen. | Section: “Methods”; Sub-Section: “Discounting of health effects and costs” |
| **Selection of outcomes** | 11 | Describe what outcomes were used as the measure(s) of benefit(s) and harm(s). | Section: “Methods”; Sub-Section: “Health Outcomes” |
| **Measurement of outcomes** | 12 | Describe how outcomes used to capture benefit(s) and harm(s) were measured. | Section: “Methods”; Sub-Section: “Health Outcomes” |
| **Valuation of outcomes** | 13 | Describe the population and methods used to measure and value outcomes. | Section: “Methods”; Sub-Sections: “Decision Problem”, “Health Outcomes” |
| **Measurement and valuation of resources and costs** | 14 | Describe how costs were valued. | Section: “Methods”; Sub-Section: “Costs” |
| **Currency, price date, and conversion** | 15 | Report the dates of the estimated resource quantities and unit costs, plus the currency and year of conversion. | Section: “Methods”; Sub-Sections: “Costs”, “Cost-effectiveness of faster recruitment to the RECOVERY Trial” |
| **Rationale and description of model** | 16 | If modelling is used, describe in detail and why used. Report if the model is publicly available and where it can be accessed. | Section: “Methods”; Sub-Sections: “Decision Problem”, “Clinical pathways and decision tree”; Figure 1 |
| **Analytics and assumptions** | 17 | Describe any methods for analysing or statistically transforming data, any extrapolation methods, and approaches for validating any model used. | Section: “Methods”; Sub-Sections: “Aims and Hypothesis” Paragraph 2, “Clinical pathways and decision tree” Paragraph 3, “Health Outcomes” Paragraphs 3 and 4, “Costs” Paragraphs 2 and 3 |
| **Characterising heterogeneity** | 18 | Describe any methods used for estimating how the results of the study vary for subgroups. | Section: “Methods”; Sub-Section: “Health Outcomes” Paragraph 2, “Costs” Paragraph 5 |
| **Characterising distributional effects** | 19 | Describe how impacts are distributed across different individuals or adjustments made to reflect priority populations. | Not reported |
| **Characterising uncertainty** | 20 | Describe methods to characterise any sources of uncertainty in the analysis. | Section: “Methods”; Sub-Section: “Probabilistic Sensitivity Analysis (PSA)”, Table S2 (Supporting Information 3) |
| **Approach to engagement with patients and others affected by the study** | 21 | Describe any approaches to engage patients or service recipients, the general public, communities, or stakeholders (such as clinicians or payers) in the design of the study. | Not applicable |
| **Results** |  |  |  |
| **Study parameters** | 22 | Report all analytic inputs (such as values, ranges, references) including uncertainty or distributional assumptions. | Table 2, Table 3, Table 4, Table S2 (S3 Supporting Information) |
| **Summary of main results** | 23 | Report the mean values for the main categories of costs and outcomes of interest and summarise them in the most appropriate overall measure. | Table 5, Table 6, Table 7 |
| **Effect of uncertainty** | 24 | Describe how uncertainty about analytic judgments, inputs, or projections affect findings. Report the effect of choice of discount rate and time horizon, if applicable. | Section: “Results”; Sub-Section: “Sensitivity analysis”, Figure 2, Figure 3 |
| **Effect of engagement with patients and others affected by the study** | 25 | Report on any difference patient/service recipient, general public, community, or stakeholder involvement made to the approach or findings of the study | Not applicable |
| **Discussion** |  |  |  |
| **Study findings, limitations, generalisability, and current knowledge** | 26 | Report key findings, limitations, ethical or equity considerations not captured, and how these could affect patients, policy, or practice. | Section: “Discussion” |
| **Other relevant information** |  |  |  |
| **Source of funding** | 27 | Describe how the study was funded and any role of the funder in the identification, design, conduct, and reporting of the analysis | Section: “Declarations” |
| **Conflicts of interest** | 28 | Report authors conflicts of interest according to journal or International Committee of Medical Journal Editors requirements. | Section: “Declarations” |
