# Supplementary material for: Improving patient recruitment to randomised trials can be cost-effective: A case-study of dexamethasone from the RECOVERY trial
Source: PLoS One. 2025 Apr 1;20(4):e0314593. doi: 10.1371/journal.pone.0314593 (PMC11961003; doi:10.1371/journal.pone.0314593)
Supplement: S2 Table — (DOCX) [file pone.0314593.s002.docx]

**S3 Supporting Information. Inputs for probabilistic sensitivity analysis (PSA)**

*S2 Table. Inputs for probabilistic sensitivity analysis (PSA)*

| Input | Source | Range | Mean | Standard error | Alpha | Beta | Distribution |
| --- | --- | --- | --- | --- | --- | --- | --- |
| *Probability inputs* |  |  |  |  |  |  |  |
| Proportion of patients who can have dexamethasone | [1] | (0,1) | 0.8277 | 0.0036 | 9354.17 | 1947.83 | Beta |
| P(acute hospital ward) | [1] | (0,1) | 0.2389 | 0.0053 | 1534.76 | 4889.24 | Beta |
| P(non-invasive ventilation) | [1] | (0,1) | 0.6044 | 0.0061 | 3882.40 | 2541.60 | Beta |
| P(invasive ventilation) | [1] | (0,1) | 0.1567 | 0.0045 | 1006.84 | 5417.16 | Beta |
| P(death\|acute hospital ward, Dexamethasone) | [1] | (0,1) | 0.1657 | 0.0166 | 82.83 | 417.17 | Beta |
| P(non-invasive ventilation\|acute hospital ward, Dexamethasone) | [1] | (0,1) | 0.0319 | 0.0079 | 15.97 | 484.03 | Beta |
| P(invasive ventilation\|acute hospital ward, Dexamethasone) | [1] | (0,1) | 0.0180 | 0.0059 | 8.98 | 491.02 | Beta |
| P(death\|acute hospital ward, No Dexamethasone) | [1] | (0,1) | 0.1325 | 0.0105 | 136.87 | 896.13 | Beta |
| P(non-invasive ventilation\|acute hospital ward, No Dexamethasone) | [1] | (0,1) | 0.0445 | 0.0064 | 45.96 | 987.04 | Beta |
| P(invasive ventilation\|acute hospital ward, No Dexamethasone) | [1] | (0,1) | 0.0290 | 0.0052 | 29.97 | 1003.03 | Beta |
| P(death\| non-invasive ventilation, Dexamethasone) | [1] | (0,1) | 0.2064 | 0.0113 | 263.79 | 1014.21 | Beta |
| P(invasive ventilation\|non-invasive ventilation, Dexamethasone) | [1] | (0,1) | 0.0790 | 0.0075 | 100.92 | 1177.08 | Beta |
| P(death\| non-invasive ventilation, No Dexamethasone) | [1] | (0,1) | 0.2170 | 0.0081 | 564.78 | 2038.22 | Beta |
| P(invasive ventilation\|non-invasive ventilation, No Dexamethasone) | [1] | (0,1) | 0.1071 | 0.0061 | 278.89 | 2324.11 | Beta |
| P(death\| invasive ventilation, Dexamethasone) | [1] | (0,1) | 0.2932 | 0.0089 | 763.23 | 1839.77 | Beta |
| P(death\| invasive ventilation, No Dexamethasone) | [1] | (0,1) | 0.4143 | 0.0097 | 1078.55 | 1524.45 | Beta |
| P(long COVID; population total) | [9] | (0.1256, 0.1309) | 0.1282 | 0.0006 | 35077.87 | 238539.13 | Beta |
| P(long COVID; ICU all) | [9] | (0.249, 0.2754) | 0.2623 | 0.0008 | 71769.74 | 201847.26 | Beta |
| *Utility inputs* |  |  |  |  |  |  |  |
| Disutility of COVID-19 Infection | [10, 12] | (0, 0.95) | 0.27 | 0.3 | 0.32 | 0.87 | Beta |
| Disutility of Hospitalisation (Acute ward, no ventilation received) | [10, 12] | (0, 1) | 0.11 | 0.3 | 0.01 | 0.08 | Beta |
| Disutility of Hospitalisation (Non-invasive ventilation received) | [10, 12] | (0, 0.96) | 0.36 | 0.3 | 0.56 | 1.00 | Beta |
| Disutility of Hospitalisation (Invasive ventilation received) | [10, 12] | (0.03, 0.99) | 0.56 | 0.3 | 0.97 | 0.76 | Beta |
| Disutility of long COVID | [10, 13] | (0.23, 0.35) | 0.29 | 0.0306 | 63.43 | 155.29 | Beta |
| *Cost inputs* |  |  |  |  |  |  |  |
| Unit cost of GP appointment (long COVID patients) | [20] | (£21.5, £64.5) | £39.23 | £10.97 | 12.79 | 3.07 | Gamma |
| Daily cost of staying in acute hospital ward | [16, 19] | (£500, £1250) | £748.41 | £191.33 | 15.30 | 48.91 | Gamma |
| Daily cost of non-invasive ventilation (oxygen) | [16, 19] | (£800, £1800) | £1394.23 | £255.10 | 29.87 | 46.68 | Gamma |
| Daily cost of invasive ventilation | [16, 19] | (£800, £4800) | £1753.94 | £867.35 | 4.09 | 428.91 | Gamma |
| Daily cost of providing 6mg of dexamethasone | [17] | (£0.21, £0.27) | £0.23 | £0.02 | 196.62 | 0.01 | Gamma |
| Annual salary cost of research nurse (Grade 5)* | [20, 21] | (£35097, £43958) | £39840.77 | £2260.46 | 310.64 | 128.25 | Gamma |

* The salary cost of research nurse is varied by -+4 spinal points, i.e. from Spinal Point 20 (Band 5), i.e. gross annual salary of £26,970 to Spinal Point 28 (Band 6), i.e. gross annual salary of £33,779. The lower and upper salary costs have considered the salary oncosts (21,22).
