# Supplementary material for: Improving patient recruitment to randomised trials can be cost-effective: A case-study of dexamethasone from the RECOVERY trial
Source: PLoS One. 2025 Apr 1;20(4):e0314593. doi: 10.1371/journal.pone.0314593 (PMC11961003; doi:10.1371/journal.pone.0314593)
Supplement: S1 File — (DOCX) [file pone.0314593.s003.docx]

**S2 Supporting Information. Estimation of the input probabilities using the results of the RECOVERY trial**

- The randomisation point of the hospitalised COVID-19 patients to the RECOVERY trial was assumed to perfectly represent the initial decision node at any hospital in real clinical settings.
- The probabilities associated with “admission” can be found in *Table 1* of the research article of the RECOVERY trial, which was also used to estimate all input probabilities but long COVID [1].
- The probabilities of the health outcomes related to invasive ventilation at the point of entry/randomisation can be found in *Figure 3* of the research article. For instance, 95 out of 324 invasively ventilated patients (at the randomisation point) died in the case of dexamethasone (P (death | invasive ventilation, Dexamethasone) = 0.293).
- The remaining probabilities were manually computed according to the available data from the RECOVERY trial.

For example, for the dexamethasone arm, the following steps were followed:

*Step 1:* 365 out of 1279 COVID-19 inpatients receiving non-invasive ventilation at randomisation, died or/and received invasive ventilation (Figure S2, Supplementary Appendix).

*Step 2:* 298 out of 1279 inpatients receiving non-invasive ventilation at randomisation, died (*Figure 3*, research article).

*Step 3:* From *Figure 3*, 89 out of 501 inpatients receiving no ventilation at randomisation, died.

*Step 4:* From *Table 2*, 25/501 inpatients in acute wards went for non-invasive ventilation or/and invasive ventilation later on (p=0.05). 20 received non-invasive ventilation and 9 received invasive ventilation. According to the rules of probability, 5 of them went straightaway to invasive ventilation ((P (invasive ventilation | acute hospital ward, Dexamethasone) = 0.018), and 16 received non-invasive ventilation immediately ((P (non-invasive ventilation | acute hospital ward, Dexamethasone) = 0.032).

*Step 5:* Since nine patients not receiving ventilation at randomisation received invasive ventilation, 101 out of 1279 patients receiving non-invasive ventilation at randomisation received invasive ventilation, according to *Table 2* of the research article. Therefore, ((P (invasive ventilation | non-invasive ventilation, Dexamethasone)) = 0.079.

*Step 6:* Considering Steps 1, 2 and 5, as well as the rules of probability, 34 out of 1279 inpatients, receiving non-invasive ventilation at randomisation, received invasive ventilation and died. Therefore, from Step 2, 264 out of 1279 inpatients receiving non-invasive ventilation at randomisation, died, without receiving any invasive ventilation. Therefore, ((P (death | non-invasive ventilation, Dexamethasone) = 0.206.

To summarise:

- 264 out of 1279 inpatients receiving non-invasive ventilation at randomisation, died, without receiving any invasive ventilation.
- 34 out of 1279 inpatients receiving non-invasive ventilation at randomisation, died, after having received invasive ventilation
- 67 out of 1279 inpatients receiving non-invasive ventilation at randomisation, survived, after having received invasive ventilation
- 914 out of 1279 inpatients receiving non-invasive ventilation at randomisation, surviving, without receiving any invasive ventilation. ((P (survival | non-invasive ventilation, Dexamethasone) =0.715.

*Step 7:* Given the yielded probabilities, P(death| acute hospital ward, dexamethasone) was computed manually.

- From Figure 3, we know that 89 out of 501 patients receiving no ventilation, eventually died.
- Patient deaths given no ventilation at randomisation, followed by non-invasive ventilation= 16 * ( P (death | non-invasive ventilation, Dexamethasone) = 0.206) = 3 (see Step 4).
- Patient deaths given no oxygen at randomisation, followed by invasive ventilation= 9 * ((P (death | invasive ventilation, Dexamethasone) = 0.293) = 3 (see Step 4).
- Therefore, P (death | acute hospital ward, dexamethasone) = 0.166 and P (survival | acute hospital ward, Dexamethasone) = 0.784, the sum of which is indeed equal to 0.95 (see Step 4).
- The same computation process was followed for the No Dexamethasone group.
